# Rapid population growth and high management costs have created a narrow window for control of introduced hippos in Colombia

**DOI:** 10.1038/s41598-023-33028-y

**Published:** 2023-04-16

**Authors:** Amanda L. Subalusky, Suresh A. Sethi, Elizabeth P. Anderson, Germán Jiménez, David Echeverri-Lopez, Sebastián García-Restrepo, Laura J. Nova-León, Juan F. Reátiga-Parrish, David M. Post, Ana Rojas

**Affiliations:** 1 Department of Biology, University of Florida, Gainesville, FL USA; 2 Fisheries, Aquatic Science, and Technology Laboratory, Alaska Pacific University, Anchorage, Alaska USA; 3 Department of Earth and Environment and Institute of Environment, Florida International University, 11200 SW 8Th St, Miami, FL USA; 4 Departamento de Biología, Pontificia Universidad Javeriana, Carrera 7 No. 43-82, Edificio Jesús Emilio Ramírez, Bogotá, Colombia; 5 Corporación Autónoma Regional de Las Cuencas de los Ríos Negro Y Nare (CORNARE), Carrera 59 44-48, El Santuario, Antioquia, Colombia; 6 Departamento de Ciencias Biológicas, Universidad de los Andes, Carrera 1 No. 18A-10, Bogotá, Colombia; 7 Department of Ecology and Evolutionary Biology, Yale University, 165 Prospect Street, New Haven, CT USA; 8 Instituto de Investigación de Recursos Biológicos Alexander von Humboldt, Bogotá D.C., Colombia

**Keywords:** Invasive species, Population dynamics, Ecological modelling

## Abstract

The introduction of hippos into the wild in Colombia has been marked by their rapid population growth and widespread dispersal on the landscape, high financial costs of management, and conflicting social perspectives on their management and fate. Here we use population projection models to investigate the effectiveness and cost of management options under consideration for controlling introduced hippos. We estimate there are 91 hippos in the middle Magdalena River basin, Colombia, and the hippo population is growing at an estimated rate of 9.6% per year. At this rate, there will be 230 hippos by 2032 and over 1,000 by 2050. Applying the population control methods currently under consideration will cost at least 1–2 million USD to sufficiently decrease hippo population growth to achieve long-term removal, and depending on the management strategy selected, there may still be hippos on the landscape for 50–100 years. Delaying management actions for a single decade will increase minimum costs by a factor of 2.5, and some methods may become infeasible. Our approach illustrates the trade-offs inherent between cost and effort in managing introduced species, as well as the importance of acting quickly, especially when dealing with species with rapid population growth rates and potential for significant ecological and social impacts.

## Introduction

Multiple factors interact to determine the likelihood that an introduced species will become established in a new environment^1,2^. Social perceptions of an introduced species can play an important role in determining their future, and public support for an introduced species can allow and even facilitate their establishment^3^. Social responses and adaptations to introduced species may play out at different temporal and spatial scales than ecological impacts, leading to “social-ecological mismatches”^4^. For example, free-ranging horses, parakeets, and camels are all examples of introduced species that engender a large degree of international human interest and concern, but that can cause significant ecological impacts at local scales^4,5^. For species such as these, positive social perceptions can constrain management decisions, which often must be made on longer time scales, and may lead to establishment or growth of species populations that ultimately have pronounced ecological consequences. These mismatches can make it difficult to predict the future costs of current decisions, and they can generate controversy over management of introduced species^6^. These dynamics may be particularly compounded in situations in which a delayed response to a species introduction leads to cost-prohibitive monitoring and management solutions, further constraining management options and ultimately increasing the likelihood of species establishment^7^.

The introduction of hippos (*Hippopotamus amphibius*) into the wild in Colombia illustrates the complex ways in which social and ecological drivers can shape the trajectory of an introduced species^8–11^. Characteristics of hippos’ life history yield potential for a rapid population growth rate, which has enabled them to establish a growing population in the Magdalena River basin. Hippos can strongly impact the environment—some effects are already apparent, and these are likely to become more pronounced in the coming decades^10^. However, prevailing social perception of the introduction has precluded culling, leading to the use of expensive and less effective methods of population control, effectively leading to exponential population growth. Hippos have now been declared an invasive species in Colombia by the Ministry of the Environment and Sustainable Development, leading the government to develop management plans aimed to reducing their distribution on the landscape^12,13^. However, these plans must account for ecological, social, and financial constraints on the ground. Herein, we provide a short overview of the history of the introduction, and we provide a population-model based assessment of the cost and efficacy of different management strategies for this population.

**Figure 1:**
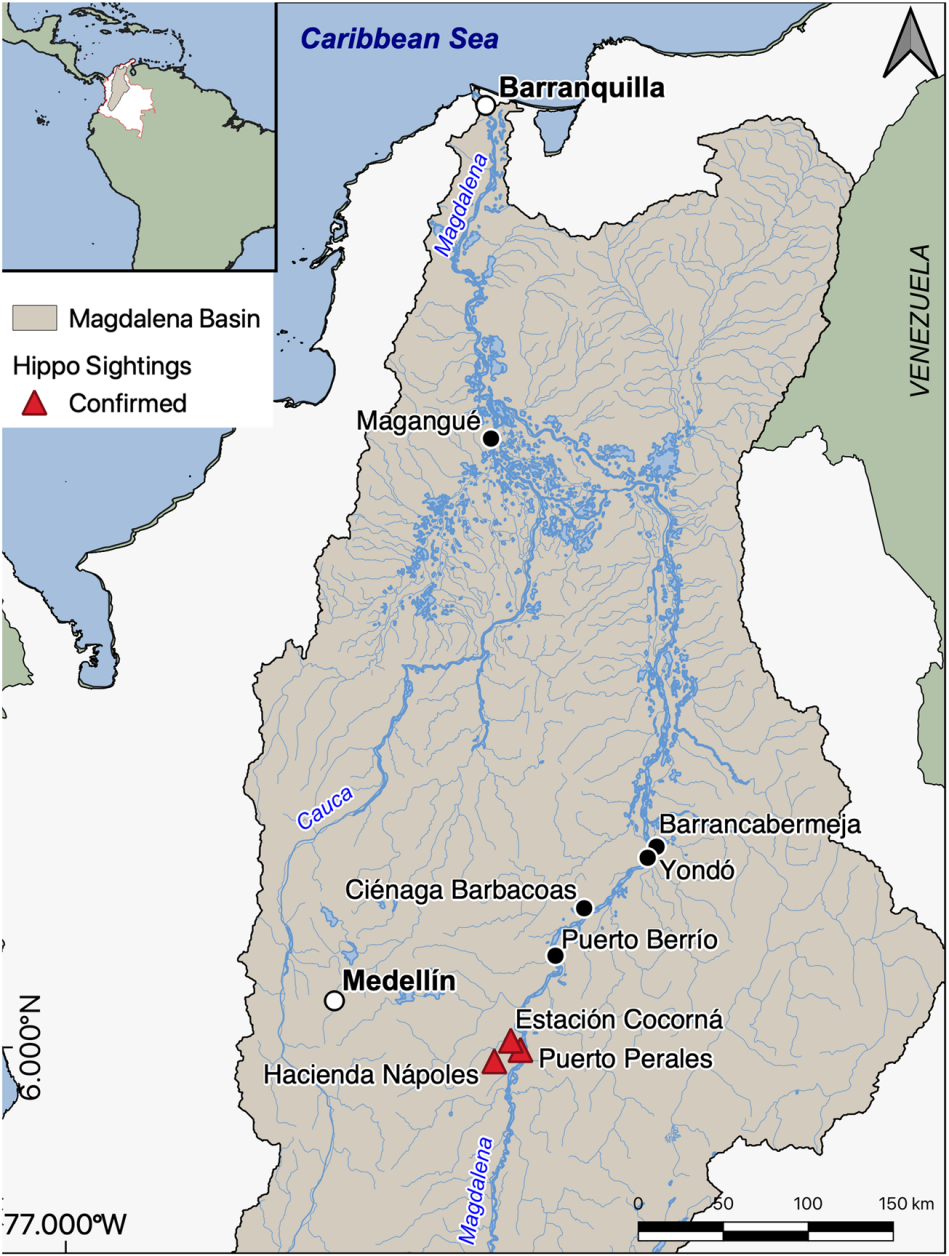
A map of the middle Magdalena River basin, with locations of the original hippo release site (Hacienda Nápoles) and confirmed hippo observations. Anecdotal accounts of hippo observations have occurred at Ciénaga Barbacoas and Magangué. The map was made by E. Bayer in QGIS, Version 3.10.5-A Coruña, https://qgis.org/en/site/forusers/download.html.

**Figure 2:**
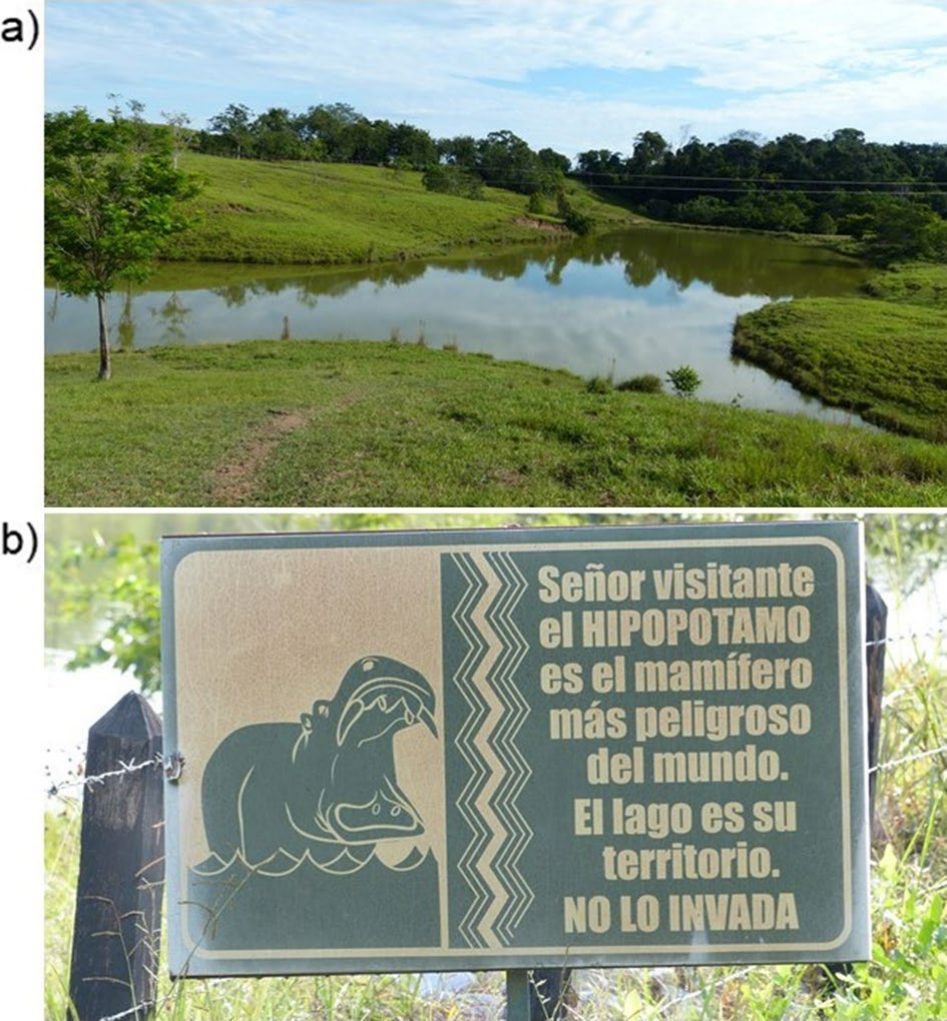
Photos from the hippo introduction site at Hacienda Nápoles. (**a)** A cattle pond near Hacienda Nápoles where three hippos reside. (**b)** A sign located near a cattle pond by Hacienda Nápoles warning visitors that hippos are considered the most dangerous animal in the world, the lake is their territory, and it should not be entered.

An initial population of 4 hippos (3 females and 1 male) were imported to Colombia in 1981 from a zoo in the United States for Pablo Escobar’s private zoo at Hacienda Nápoles (Figs. 1, 2). After Escobar’s death in 1993, the zoo was closed, but the hippos were left on site due to challenges with transporting them to new habitats, and they began to reproduce and disperse into the surrounding landscape. In 2009, when there were an estimated 28 hippos in the population, an aggressive male hippo was culled by environmental authorities, prompting an outcry from national and international animal rights activists. A judicial ruling banned shooting of hippos, amidst objections by Colombian scientists. In lieu of culling, surgical sterilization and containment were adopted as the primary management strategies to control hippos^14^. Male hippo sterilization began in 2011; however, to date only ten males have been treated, partly due to the costs and challenges associated with this procedure^15^. New efforts are exploring the use of chemical sterilization with GonaCon Immunocontraceptive Vaccine (NWRC, Fort Collins, Colorado, USA), which has been used to induce contraception in many mammalian species^16,17^. Since 2021, 24 individuals have been treated. Repeated use can lead to long-term infertility in some species, although this has yet to be proven in hippos. Recent management recommendations by environmental authorities have suggested humane euthanasia as an option in certain situations, although public opinion is still strongly divided on this approach.

Since their release to the wild, the hippo population has continued to grow at an estimated rate of at 7–14.5% per year^8,9^. As of 2020, there were an estimated 75 hippos located in the middle Magdalena River basin, although formal field-based population censuses are still ongoing. There were approximately 58 hippos located in partial captivity near Hacienda Nápoles, and an estimated 17 hippos that had dispersed to the north along the Magdalena River and nearby tributaries (Fig. 1). Confirmed observations have been documented in Puerto Perales and Estación Cocorná. Anecdotal observations have been reported nearly 400 km away in Magangué^9^. If left unmanaged, the Colombian hippo population could reach > 1,000 individuals and occupy the entire Magdalena River basin within the next 50 years^9^, with potential to lead to novel social-ecological system configuration^8,10^.

Hippos are a charismatic animal, and their introduction to Colombia has attracted global attention and engendered a range of social responses. Colombian and international scientists have expressed the need for a concerted management strategy to control the population growth and dispersal of hippos, citing concerns about the potential for human-wildlife conflict, as well as for protection of native flora and fauna in the region^8–11^. Population modelling has suggested the only course of action that could likely lead to the elimination of wild hippos in Colombia is to institute culling^9^. In contrast, a local and international animal rights movement has developed to protect the hippos, with a 2021 lawsuit filed in the U.S. declaring the Colombian hippos as “interested persons” in the case^18^. Local inhabitants of the middle Magdalena River basin are perhaps the most directly impacted, given the proximity of the hippos to agricultural lands and towns, as well as extensive use of the river for livelihoods including fishing. At least two residents of the middle Magdalena basin—one farmer and one fisherman—have been attacked and injured by hippos since 2019. However, hippos also have become a local tourist attraction in the region that provides commercial benefits to some residents, including the recent rise of boat tours to view hippos. The hippos are also closely associated with the complex legacy of Pablo Escobar, and both positive and negative perceptions of his role in the region likely influence how people view them. Anecdotal reports suggest interest in the hippos has led to occurrences of human-mediated dispersal, which may become a growing concern the longer the population persists.

Incidences of human-wildlife conflict and the potential ecological effects of this introduction are likely to increase as the hippo population grows^19,20^. By the time either social or ecological concerns become more pronounced, it may be infeasible to remove hippos from the landscape given their long lifespans, high population growth rate, and potential for widespread dispersal. Furthermore, potential management costs are likely to increase substantially the longer the situation unfolds. Environmental authorities with CORNARE (Corporación Autónoma Regional de las Cuencas de los Ríos Negro y Nare), in the region in which Hacienda Nápoles is located, continue to work towards containment and sterilization of hippos with available staffing and financial resources. The Ministerio de Ambiente y Desarrollo Sostenible has recently launched an alliance between scientists and local communities to develop a national management strategy for the hippos^21^. The cost and efficacy of strategies to control the Magdalena hippo population are poorly understood and may ultimately constrain the feasibility of population management. Addressing this knowledge gap and identifying necessary resources to effectively control the introduced hippo population presents a key management priority for the Magdalena River ecosystem.

This paper represents a collaborative effort by Colombian and U.S. scientists and resource managers, with expertise in both hippos’ introduction in Colombia and their native range in Africa. Our aim is to provide environmental authorities in Colombia with an objective quantification of proposed management strategies, which has been identified as a key information need by national and international environmental authorities^22^. We developed a population model for the Magdalena River hippos to assess the cost and efficacy of different management strategies for control of this introduced population. Our objectives were to i) assess the current population abundance and growth rate of the Magdalena hippo population, ii) quantify the impact of different management interventions on hippo survival and population fertility, and iii) estimate the cost and time to remove the Magdalena hippo population from the landscape across different management strategies.

## Results

### Projection modelling

**Figure 3:**
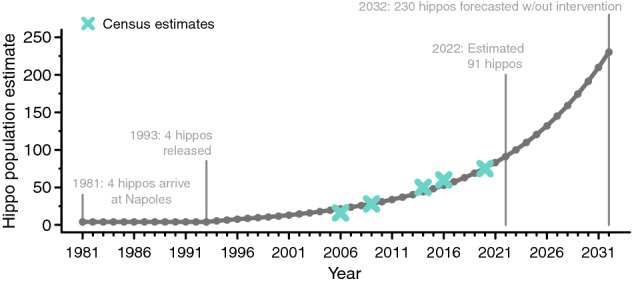
Colombian hippopotamus introduction timeline and population growth estimate.

Our population estimates fit well with observed Colombian hippo census numbers and indicate that total population growth is currently approximately 9.6% per annum (Fig. 3). The standing population of introduced hippos in the Magdalena River basin was estimated at 83 hippos by the end of 2021 and 91 hippos by the end of 2022, and it is projected to increase up to 230 hippos within the next decade if growth remains unchecked and continues a density-independent path. This rate is intermediate to previously published estimates (7–11%^8^, 14.5%^9^), and our estimates may be conservative.

Elasticities indicate that female Colombian hippo population growth is most sensitive to changes in survival (sum of survival elasticities for survival ages 1 to 45 = 0.92), and relatively less sensitive to reductions in fertilities (sum of elasticities for all age-specific fecundity parameters combined = 0.08). For example, female survival across all ages would need be reduced proportionately by 9.5% to stabilize the population at zero growth (i.e. $\lambda =1.0$), whereas fertility rates for females would need be reduced proportionately by 77% to yield the same effect. To reduce female fertility by this amount through reduction of fertile males, the number of current fertile males would have to be reduced by 93%.

### Management interventions

**Figure 4:**
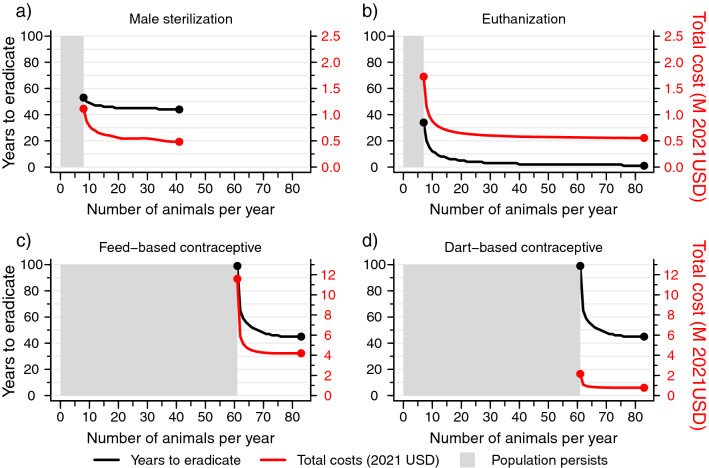
Estimated time to eradication (left y-axis) and total costs (right y-axis, 2021 USD) for different management interventions to control introduced hippos in the Magdalena River basin, Colombia. Management interventions (a-d) assume a fixed numbers of animals (x-axis) are addressed each year starting from 2022 until eradication is achieved. The minimum number of animals that could be addressed annually and still lead to hippo eradication differs across control strategies; treating fewer than this minimum number of hippos annually will lead to long term hippo population persistence (gray polygons). The standing stock of hippos may drop below the target annual animal treatment number under a given management intervention; thus, costs are calculated as the realized total number of animals addressed to achieve eradication under a given intervention approach multiplied by a per-individual treatment cost.

Among the legally available management options to control Colombian hippos, male sterilization is the most cost-effective option currently available (Fig. 4a; Table 1). The most rapid eradication of the population occurs if all males are sterilized in the first year of management intervention. This would entail operating on an estimated 42 males at large in the population as of the start of 2022, at a cost of 0.53 M USD. However, as few as 9 males per year sterilized would still lead to population eradication in 52 years, although at a higher total cost in current year dollars of 1.14 M USD. Annual contraceptive dosing with either feed or darts are both expensive options that would take decades and considerable effort to control the hippo population (Fig. 4c-d; Table 1). For example, because it is not possible to reliably target specific sexes in administering contraceptives in the field, it would require treatment of the entire estimated current population of hippos (n = 83 at the start of 2022) for 45 years consecutively to achieve eradication, at a current dollar total cost of 0.85 M USD for dart-based treatments or 4.59 M USD for feed-based treatments.

**Table 1:** Cost minimizing and annual effort minimizing strategies to control introduced hippos in the Magdalena River, Colombia starting from present time or delaying action for a decade. We used the hippopotamus population at the close of 2021, n = 83, to represent the starting population size for management interventions commencing in 2022. Similarly, we used the population abundance at the close of 2031, n = 210, for management interventions commencing a decade out in 2032. Years to eradicate indicates the total years of treatments needed to eradicate the entire population (males and females) once management interventions have commenced.

Management strategy	Cost minimizing						Effort minimizing					
	Starting from present time			Delaying action for 10 years			Starting from present time			Delaying action for 10 years		
	Years to eradicate	Animals per year	Total cost (M 2021 USD)	Years to eradicate	Animals per year	Total cost (M 2021 USD)	Years to eradicate	Animals per year	Total cost (M 2021 USD)	Years to eradicate	Animals per year	Total cost (M 2021 USD)
Male sterilization	44	42	0.53	45	105	1.34	52	9	1.14	58	21	3.98
Veterinary-assisted euthanasia	1	83	0.61	1	210	1.54	27	8	1.58	38	19	5.29
Feed-based contraceptives	45	83	4.59	45	210	11.47	84	67	9.96	95	168	30.04
Dart-based contraceptives	45	83	0.85	45	210	2.13	84	67	1.85	95	168	5.58

In contrast to sterilization or contraception, which impact population fertility and thus require significant time periods for the standing population to age and eventually expire, veterinary-assisted euthanasia affects survival and has a more rapid impact on the population (Fig. 4b; Table 1). Furthermore, veterinary-assisted euthanasia permanently removes animals from the population and does not require repeated treatments of individuals. Theoretically all of the estimated current standing stock of hippos could be euthanized in a single year at a cost of 0.61 M USD; however, commencing in 2022 as few as 8 animals per year could be euthanized and achieve eradication after 27 years at current dollar total cost of 1.58 M USD.

Delaying the start of hippo management efforts for a decade (until the start of 2032) would significantly increase the time and cost associated with population control. The total cost of cost-minimizing scenarios in current dollars increases by a factor of approximately 2.5 if action is delayed by a decade (Table 1). For example, the cost-minimizing male sterilization scenarios commencing action a decade from now in 2032 would require treating 105 males in a single year at a present dollar total cost of 1.34 M USD. Similarly, by delaying action until 2032, the minimum number of males that could be sterilized per year and still achieve eradication would increase substantially to 21 animals annually. In terms of contraceptives, cost-minimizing scenarios after a 10-year delay increase to 2.13 M USD and 11.47 M USD for dart- and feed-based dosing, respectively. Effort-minimizing scenarios for contraceptive-based treatments after a 10-year delay would require high numbers of animals treated annually (n = 168), very high total costs in current dollars (dart-based dosing: 5.57 M USD; feed-based dosing: 30.04 M USD), and it would entail inordinately long eradication times (95 years of consecutive treatments).

## Discussion

We estimate that as of the close of 2022, there were approximately 91 hippos in the middle Magdalena, and the population is growing at 9.6% per year. At this rate, there will be 230 hippos within a decade (at close of 2032) and over 1000 by 2050. Given the high population growth rate of hippos and the current number of animals, it will require significant investments of money, effort, and time to remove hippos from the wild in Colombia even if aggressive interventions start immediately. Delaying management actions for a decade will increase minimum costs by a factor of 2.5, and some interventions may become infeasible.

If the population control methods we examined here are applied immediately and at large scales, it would require a minimum of 0.5–4.6 million USD to remove hippos from the Magdalena River if cost-minimizing approaches are implemented (Table 1). Instead, if annual effort-minimizing approaches are implemented, the total costs would more than double (1.14—9.96 million USD). There are significant challenges associated with each management approach, and social and ecological considerations will need to be weighed to determine the best path forward. However, the longer interventions are delayed, the more difficult and expensive they will become. In another decade, minimum costs will increase to 1.3–11.5 million USD, and the logistics of treating the population would become increasingly difficult with an estimated 210 hippos at large by the close of 2031. Furthermore, action delays that lead to increasing hippo population size and length of time on the landscape could also increase the possibility of natural or human-mediated dispersal into other systems.

Male sterilization is the least expensive way to control the hippo population among the management interventions considered here. However, this process entails hippos being captured, anesthetized, transported by helicopter, and surgically operated upon; thus, it is very challenging and can be dangerous for both the people and hippos involved. Further, male sterilization must be done on many animals every year in order to be effective as a population control strategy. This management intervention also fails to remove hippos from the landscape, and given their long lifespan, would result in hippos remaining in the middle Magdalena for several more decades at least. Therefore, this approach alone may not reduce risks hippos pose to people who use the river for their livelihoods or to native biodiversity in the Magdalena River basin^8^. Veterinary-assisted euthanasia is the second most cost-effective method of population control, although it is still relatively expensive given the requirement to capture and anesthetize hippos first. However, it is the most effective way to remove hippos from the landscape.

Dart-based contraception was the third most cost-effective method, as the drugs are inexpensive. However, this method likely must be applied every year and for many years consecutively. Research in other taxa has suggested injection of GonaCon as a contraceptive may promote long-term infertility after several rounds, although this has yet to be demonstrated in hippos^23^. It would also be challenging to use on hippos outside of captivity, as capture is typically needed to ensure intramuscular injection. Oral-based contraception, which is frequently used in captive populations, was the most expensive of the options. It also would be very challenging to apply to hippos outside of Hacienda Nápoles, suggesting another method would be needed for these individuals, or they would need to be captured and relocated to captivity. Both dart- and oral-based forms of contraception would also leave hippos on the landscape for many years to come, with associated risks to social and ecological systems.

Elasticity analyses suggest that targeting young female hippos is the most effective way to decrease the hippos’ population growth rate; however, management intervention options to target this demographic group through mortality or fecundity control are limited. While theoretically these animals could be preferentially targeted through birth control or veterinary-assisted euthanasia, it is difficult to identify hippo age and sex without capturing the animal first. Additional options include preferential relocation of young females to zoos when possible, and surgical sterilization of females, although female surgical sterilization is even more complex and dangerous than surgical sterilization of males.

We did not explicitly incorporate the spatial structure of the hippo population in our estimates, which may further compound the challenge and cost of management, although our management cost estimates did include higher costs associated with hippos at large outside of Hacienda Nápoles. Approximately 53% of the current estimated population lives within Hacienda Nápoles, 24% lives in nearby lakes, and 23% lives in the mainstem Magdalena River or much further downstream. It is likely that as hippos spread further into the landscape, the logistical challenges in locating and handling hippos will increase significantly, and some population control approaches, such as feed-based contraceptives, may become nearly impossible to implement^24^. Other approaches that require animal capture and sedation, such as male sterilization and veterinary-assisted euthanasia, will be much more logistically challenging, particularly as individuals continue to disperse, and these dispersal events will likely continue to seed population growth in the surrounding areas. Ensuring containment of hippos within Hacienda Nápoles to prevent further escapes will improve chances of managing the population. However, containment itself is costly and challenging, in addition to the management costs we estimate here. Furthermore, some people disagree with containment of the hippos, arguing they should be allowed to stay free, suggesting even this management option is not without potential social constraints (Anderson et al. In prep).

It is possible that some other driver could act to decrease population growth, or even lead to a population crash, such as the emergence of a zoonotic epidemic or inbreeding effects from a strong founder effect that reduces genetic diversity in the population. Many invasions follow a boom-bust dynamic^25^. Hippos in particular can be heavily impacted by anthrax outbreaks and can be sensitive to starvation^26^. The population in Colombia started with only 4 individuals, only one of which was male, suggesting that the relatively low genetic diversity in the founding population and subsequent inbreeding could lead to reduced genetic fitness^27,28^, although effects of inbreeding are not always as strong as predicted in wild populations^29^. The Colombian hippo population is currently growing at a rate similar to that of healthy, resource-unlimited populations within their native range. Furthermore, the central Magdalena basin lacks the pronounced dry season that often acts to limit population growth in hippos’ native range through periods of drought and associated hippo health declines^10^. Given the rapid rate of annual population growth and the uncertainty of any future limits on this growth, it is critical that management practices are adopted quickly.

The cost of interventions is extremely high and exceeds the current available funding for hippo management in the Magdalena River. Furthermore, our results are likely conservative because they assume the first and last hippo to be treated are the same cost, whereas search costs for any of the treatment options may increase as the hippo population declines. These costs will continue to increase over time if substantial interventions are delayed. A 10-year delay will result in 2.5 times increase in minimum costs for each management approach. Culling hippos without the considerations required for veterinary-assisted euthanasia could provide a more cost-effective management approach than those we explored here. However, it is currently not a legal option in Colombia and would likely engender strong objections by some parties^18^. Additional management resources beyond those that are currently available will need to be mobilized to support introduced hippo management in Colombia, which may in turn lead to diversion of resources from other conservation efforts in the region, to the potential detriment of other flora and fauna in the Magdalena River, a riverine biodiversity hotspot^30–33^. International funding could be sought to assist with the hippos’ management, but this would likely divert resources that could otherwise go to bolster research and conservation efforts in the species’ natural range.

Social and financial constraints have limited management options for hippos in the Magdalena since the start of their introduction to the wild in the late 1990s-early 2000s. These constraints allowed the hippo population to begin growing at a rapid rate that has now increased the cost and difficulty of management options. It will now take at least 1–2 million USD to sufficiently decrease hippo population growth, and depending upon what management strategy is selected, there may still be hippos on the landscape for 50–100 years. Increased population size and persistence will likely increase the potential for natural and human-mediated dispersal, so hippos are likely to continue to expand throughout the Magdalena River basin and possibly into other regions as well. These hippos will continue to influence social and environmental systems during this time, with consequences for goods and services provided for human communities as well as for biodiversity. Social and ecological impacts of this introduction are still nascent but are likely to become much more pronounced with increased population growth. Even if social perceptions about appropriate management approaches shift over the coming years, some management options may become infeasible in the future given the current rapid annual rate of population growth. This case study illustrates the complex interplay between social, financial, and ecological dynamics that can influence the trajectory of species invasions and suggests early stakeholder communication and outreach is a critical component of invasive species management plans^4,34^. Our results highlight the urgent need for sufficient funding to undertake a large-scale effort to control the growth of the Colombian hippo population while it is still feasible.

## Methods

### Study area

Hacienda Nápoles is located in the middle Magdalena River Basin, near the towns of Doradal and Puerto Triunfo and roughly halfway between Colombia’s two largest metropolitan areas of Bogotá and Medellín. The region surrounding Hacienda Nápoles was historically dominated by tropical, moist forest. Much of the landscape has been converted to human settlement and agriculture, although the middle Magdalena remains an important region for native biodiversity^35,36^. Many of the hippos in the region occupy small cattle ponds along riparian corridors during the day and move inland at night to graze in surrounding grasslands (Fig. 2). The abundance of grazing land for livestock and the lack of dry season conditions may reduce resource limitation which, in addition to a lack of natural predators, may allow for high rates of hippo population growth^10^.

### Population model

We developed a birth-pulse age-structured Leslie matrix model to estimate the current Colombian hippo population size, assess the influence of reductions in survival versus fertility on population growth, and forecast the cost and efficacy of different management interventions to control hippos^37^. We implemented the Colombian hippo population model in the R statistical programming environment using custom scripts^38^. Full model formulae and parameter values for the model are included in Supplementary Text S1. The population model tracked females and males separately. A wide range of reproductive parameters have been documented in captivity and in the wild for hippos, suggesting they have considerable plasticity in life history traits^39–41^. Colombian hippos have low densities and abundant food and habitat in the Magdalena River ecosystem, and the source population at Hacienda Nápoles enjoys some husbandry care in partial captivity. This suggests that the combined population of introduced Colombian hippos have demographic rates that are intermediate between values demonstrated by captive hippos and values measured in field conditions in their natal African range^9^. For our Colombian hippo population model, we sourced baseline life history parameters based on values from low-density hippo populations in their native range, which would reflect low mortality and high fecundity leading to density independent growth^40^. These baseline parameters were subsequently adjusted and tailored to reflect Magdalena River hippo ecology as informed by field observations from resource managers in Colombia familiar with the introduced hippo population.

Age at first reproduction was set to 3 years for both males and females, based on values from the literature and anecdotal observations from Colombia^40^. We used a logistic growth equation to estimate increasing fecundity from 0% at age 2 to 50% of maximum fecundity at age 5 and full fecundity at age 9 through 25, followed by a linear decline in fecundity of 5% per year after age 25^40^. We set full fecundity at 0.5 indicating birth of one calf every two years, in accordance with observations from low-density populations in the field^40,42^. We assumed a 50:50 sex ratio for births, as has been typically documented in the wild (Supplementary Table S1).

Natural mortality for the Magdalena hippo population is believed to be lower than that for hippos in their native range, based on anecdotal observations and given the fact that these introduced hippos face no natural predators and little if any resource limitation^9^. We used estimated age-specific mortality rates for low-density hippo populations, with a rate of 12% for ages 0–1, 6% for ages 1–2, 3% for ages 3–30, and increasing exponentially for hippos > 30 years of age (Supplementary Table S2)^40^. We also specified sex-specific natural mortality rates, as males tend to have higher mortality rates due to territorial interactions and high rates of dispersal^39^. We adjusted mortality rates for animals above 10 years of age by increasing male mortality by 5% and decreasing female mortality by 5%^40^. We set the maximum age of hippos at 45 years for males and females^43^.

The baseline model commenced with the initial population of one male and three females kept in captivity at Hacienda Nápoles beginning in 1981, each with an assumed initial age of 10 years based upon anecdotal records. We assumed no hippos died during captivity; therefore, mortality was fixed at 0 until the first full year subsequent to their 1993 release. Similarly, breeding was modelled to commence in the first full year after their release.

We modelled population growth using a density-independent matrix model, as there is no indication that hippos will approach a carrying capacity in the Magdalena River basin in the coming decades with access to vast swaths of riverine habitat. Mean hippo densities of 35 hippos/km have been documented in rivers in their native range^44,45^. Given an estimated potential range of 625 km in the Magdalena downstream of the current southernmost observation^9^, more than 20,000 hippos could persist at this density. Population projections were compared against five hippo censuses informed by resource managers in the Magdalena region (2006: 16 hippos, 2009: 28, 2014: 50, 2016: 60, 2020: 75)^8^.

Finally, to provide insight into the impact of different management strategies on population growth rates, we calculated population growth elasticities for the female population, which provide the percentage change in population growth rate, $\lambda$, with a percentage change in a specific vital rate such as fecundity or mortality at age. We focused elasticity analysis on female hippos as the source of recruitment to the population. Elasticities were summed across survival parameters and fecundities at age to represent the collective influence of different demographic processes on female population growth^37^.

### Management interventions

We explored the efficacy of four hippo population control management options currently under consideration by Colombian resource managers: (1) male surgical sterilization, (2) veterinary-assisted euthanasia, (3) oral-based contraceptives, and (4) dart-based contraceptives. Female hippo surgical sterilization is not presently being considered due to high expense and uncertainty about the safety and efficacy of the procedure. Both male sterilization and veterinary-assisted euthanasia require capturing and anesthetizing individuals (Supplementary Table S3). We modelled the impact of male sterilization on hippo population growth by linking the number of fertile mature males to a scalar discount factor which reduced the female hippo fertility schedule. The fertility reduction factor was calculated from a saturating function based on the number of males and harem size reflecting the polygynous breeding behavior of hippos (Text S1). That is, when sufficient fertile males exist to sire the entire female population, fertility is unadjusted, whereas at intermediate numbers of fertile males, fertility is reduced linearly. We modelled veterinary-assisted euthanasia as a ‘harvest’ rate and added this as an additional source of mortality to the population model.

Contraceptives remove treated females from the breeding population on a temporary basis, and typically require repeated doses. Melengestrol acetate (MGA) can be added into feed to provide an oral contraceptive, which should be consumed daily. The majority of the current population is still within the Hacienda Nápoles borders and receives supplemental feeding, so MGA-amended feed could be provided to this portion of the population. Contraceptives also can be administered by darting, although animals typically need to be captured first to ensure intramuscular injection. GonaCon is a gonadotropin-releasing hormone (GnRH) immunocontraceptive vaccine that stimulates the production of antibodies that bind to GnRH and reduces the production of sex hormones and sexual activity^16^. A single intramuscular injection could prevent reproduction for a year or possibly longer. Repeated injections have caused longer periods of infertility in other animals^23^, although this has yet to be tested in hippos. Thus, we modelled both feed- and dart-based contraceptive dosing as being administered annually to achieve a fertility effect. Because sex is difficult to decipher visually from a distance, we modelled both oral and dart-based contraceptives as agnostic to sex. Subsequently, we discounted the baseline female fertility schedule by a scalar factor equivalent to the proportion of females dosed with contraceptives in a given year.

We assessed the efficacy of different management strategies by conducting population projection simulations in which we specified a fixed number of animals to be sterilized, euthanized, or dosed with contraceptives annually, and then applied treatments until the population (males and females combined) was eradicated. We used a grid search to determine the treatment levels associated with minimum total cost (in 2021 USD) or minimum annual effort required to achieve eradication (i.e. < 1.0 hippo remaining in the population) for each of the four management options. While cost-minimization may reflect a desirable criterion to select a hippo eradication strategy, pragmatically, the availability of staffing and labour to administer population control strategies is often constrained. Thus, we also investigated which management strategy could successfully eradicate hippos with the smallest number of animals treated annually as a proxy to reflect potential field effort limitations. Finally, to investigate the cost of waiting, we explored scenarios implementing management interventions immediately (2022) or delaying implementation for a decade. We estimated the per animal treatment costs used for management simulations in consultation with veterinary staff and resource managers familiar with hippo biology (Supplementary Table S3). All monetary values are presented in 2021 USD.

## Supplementary Information

Supplementary Information.

## Data Availability

All data analyzed for this study are included in the main manuscript and supplementary files.
