# Supplementary material for: Rapid population growth and high management costs have created a narrow window for control of introduced hippos in Colombia
Source: Sci Rep. 2023 Apr 16;13:6193. doi: 10.1038/s41598-023-33028-y (PMC10106455; doi:10.1038/s41598-023-33028-y)
Supplement: Supplementary file 1 — Supplementary Information. [file 41598_2023_33028_MOESM1_ESM.pdf]

Supplementary Information

Rapid population growth and high management costs have created a narrow window for control of introduced hippos in Colombia

Amanda L. Subalusky, Suresh A. Sethi, Elizabeth P. Anderson, Germán Jiménez, David Echeverri Lopez, Sebastián García-Restrepo, Laura J. Nova León, Juan F. Reátiga Parrish, David M. Post, Ana Rojas

Contents:

Text S1. Colombian hippopotamus Leslie matrix model.

Table S1. Colombian hippo population model parameters.

Table S2. Colombian hippopotamus projection matrix parameter values.

Table S3. Detailed estimates of cost associated with each management scenario.

Figure S1. Female fecundity impacts associated with male hippo sterilization management interventions. Calculations show fecundity adjustment factors assuming a population of 100 sexually mature females and four different values for the harem size parameter, x_f, representing the of the number of females a single male can sire in a breeding season (eq. S4).

Text S1. Colombian hippopotamus Leslie matrix model

Baseline population model

We constructed a birth-pulse age structured Leslie matrix project model which runs on an annual time step to represent Colombian hippo population dynamics. The basic population dynamics are governed as:

$$\boldsymbol{n}_{t+1} = \mathbf{A}\boldsymbol{n}_t \qquad \text{eq. S1,}$$

where $\boldsymbol{n}$ is a vector of population sizes at some time step, and $\mathbf{A}$ is the square projection matrix encompassing birth and death vital rates. The top row of the project matrix contains fecundities, f_a, which represent the per capita birth rate for a given age a, and the subsequent rows contain the fraction of age a individuals that survive to the next age $a+1$ (and time step), s_a, where no individuals survive past terminal age a^* (i.e. $s_{a^*} = 0$):

$$\mathbf{A} = \begin{bmatrix} f_1 & \cdots & \cdots & f_{a^*} \\ s_1 & 0 & 0 & 0 \\ 0 & \ddots & 0 & 0 \\ 0 & 0 & s_{a^*-1} & 0 \end{bmatrix}.$$

To accommodate management simulations which target specific sexes (e.g. male sterilization), we constructed project matrices for males and females separately, which are linked by allocating total young born in a year into females and males following a sex ratio parameter. Life history parameter values were based on information from African hippos (Martin 2005), but updated to reflect Colombian hippo biology as informed by resource managers familiar with the

introduced hippo population (Table S1). The resulting life history parameters values are consistent with those from African hippos under settings of density independent growth without resource limitation (Table S2).

We assessed the elasticity of population growth for the female population to changes in age specific survivals or fecundities to investigate which life history processes are most responsive to management interventions (Caswell 2001, Carslake et al. 2009). We calculated an elasticity matrix, $\mathbf{E}$, representing the percent change in the population growth rate at stable age distribution with a percent change in a given element in the projection matrix (i.e. either a fecundity or survival):

$$\mathbf{E} = \left[\frac{\mathbf{v}\mathbf{w}^{\mathrm{T}}}{\mathbf{v}^{\mathrm{T}}\mathbf{w}}\right] \circ \mathbf{A}\frac{1}{\lambda} \qquad \text{eq. S2,}$$

where λ, the population growth rate at stable age distribution, is the dominant eigenvalue of the projection matrix $\mathbf{A}$, and $\mathbf{v}$ and $\mathbf{w}$ are the left and right eigenvectors associated with the dominant eigenvalue. Note, "∘" indicates element-wise matrix multiplication. Because we focused elasticity analysis only on the female population, fecundities were scaled by the sex ratio (50:50) to reflect births only in females. Elasticities among age-specific survivals or fecundities can be summed to represent the influence of groups of vital rates on overall population growth. Thus, we summed elasticities across all fecundities at age and survival at age to reflect the response of female population growth to relevant management strategies. Finally, we calculated the projected percentage change in female population growth rate resulting from a given proportionate change, $\delta_{i.j}$, to the i, j^{th} component of the projection matrix as (i.e. see section 9.2.5 in Caswell, 2001):

$$\Delta\lambda = \sum_{i,j} e_{i.j}\delta_{i.j} \quad \text{eq. S3,}$$

where $e_{i.j}$ is the i, j^{th} component of the elasticity matrix. We used this to solve for the required proportionate change across all vital rates for a given demographic grouping of vital rates that would be needed to reduce female population growth rate below 1.0 (i.e. declining population growth).

Initiating the baseline model

We initiated the model commencing with the arrival of the single male and three female captive hippos at Hacienda Nápoles in 1981 (i.e. $t_0 = 1981$), assuming the initial age of each of the four hippos to be 10 years based upon feedback from resource managers in the region. However, as no hippos died during captivity, we fixed survival at 1.0 for all ages until the first full year after their release, i.e. with mortality commencing in 1994. Hippos did not breed during captivity, and we similarly modelled a delay in breeding until 1994.

Modelling management interventions

Four management options were mapped onto fertility and survival outcomes for Colombian hippos: male sterilization, oral contraception, dart-based contraception, and veterinary-assisted euthanasia. Modelling the impacts of male hippo sterilization required accommodating polygynous male breeding. Thus we linked male sterilization to female fecundity rates through a discount factor, α, that ranged from 0.0 to 1.0 following a hockey-stick

saturating function based on the number of mature fertile males at large in the population and a parameter, x_f, representing the average number of females in a given breeding male's harem:

$$\alpha = \begin{cases} 1.0 & \text{if} \quad n_{m,a>\tilde{a}} \geq n_{f,a>\tilde{a}}/x_f \\ \left(x_f n_{m,a>\tilde{a}}\right)/n_{f,a>\tilde{a}} & \quad n_{f,a>\tilde{a}}/x_f > n_{m,a>\tilde{a}} > 0 \\ 0.0 & \quad n_{m,a>\tilde{a}} = 0 \end{cases} \qquad \text{eq. S4,}$$

where $n_{f,a>\tilde{a}}$ and $n_{m,a>\tilde{a}}$, are the total number of sexually mature females and males (i.e. age greater than age at first sexual maturity, $\tilde{a}$) at large in the population. We assume that males guard exclusive harems, such that females do not switch between males during the breeding season. In this case, as long as there are at least $n_{f,a>\tilde{a}}/x_f$ fertile males, then all mature females can be successfully bred and $\alpha = 1.0$ (i.e. no female fecundity discount). Of course with no fertile males, no females breed and $\alpha = 0.0$. At intermediate numbers of fertile males then the fecundity discount factor increases linearly as a function of the harem size parameter (Fig. S1). Subsequently, fecundity rates are scaled as $\alpha \times f_a$ in the population projection matrix **A**.

Oral and dart-based contraceptives are administered annually and temporarily render female hippos as unfertile during a given year. Hippos are not readily distinguishable by sex visually, and thus we modelled contraceptive treatments as agnostic to sex. Contraceptive treatments were linked to female fertility through a fecundity discount factor, β, calculated as the proportion of the total standing population treated in a given year:

$$\beta = 1 - {n^s}/{n} \qquad \text{eq. S5,}$$

where n^s is the number of animals treated with contraceptives (either orally or dart-based) in a given year, and n is the total standing population size at the time of treatment. Subsequently, fecundity rates are scaled as $\beta \times f_a$.

Veterinary-assisted euthanasia was modelled as an additional source of mortality calculated as the proportion of the total population euthanized, γ, during a given year:

$$\gamma = {}^{n^u}/_{n} \qquad \text{eq. S6,}$$

where n^u is the number of animals to be euthanized in a year, and n is the total standing population size at the time of harvest. Subsequently, age-specific survival under culling, s_a^u, was calculated by discounting baseline survival as: $s_a^u = s_a\gamma$.

For each management strategy, we conducted simulations to identify treatment schedules which minimize total cost or annual effort (in number of animals handled per year) in eradicating the hippo population, males and females combined. We specified a fixed number of animals to be treated each simulation year and applied this until eradication was reached, investigating each population control option in isolation (oral or dart-based contraceptives, male sterilization, and veterinary-assisted euthanasia). Simulations were commenced from the start of 2022 and after a ten year delay (commencing at the start of 2032), to explore the additional cost and time required to eradicate hippos if population control efforts were delayed. We used a grid search to solve for optimal treatment levels. Per-animal costs were specified as one-off expenses for veterinary-assisted euthanasia and male sterilization, whereas costs for oral and dart-based contraceptives

dosages were charged annually as these treatments last for a single year. Values for per animal treatment costs were estimated by consulting with veterinary staff and resource managers familiar with the Colombian hippo population (D. Echeverri, pers. comm., Table S3). All management costs were tracked in present 2021USD.

Table S1: Colombian hippo population model parameters

Parameter	Value
Maximum age: age after which mortality is 100%	45 years
Age at first reproduction (females): earliest age at which females become reproductive	3 years
Age at 50% reproduction (females): age at which 50% of females are reproductive	5 years
Age at 100% reproduction (females): age at which 100% of females are reproductive	9 years
Age at first reproduction (males): earliest age at which males become reproductive	3 years
Sex ratio: ratio of females to males at birth	50%
Harem size: number of females a male can sire in a season	5 females
Fecundity: maximum number of calves per year per female	0.5 calves

Table S2. Colombian hippopotamus projection matrix parameter values.

Age	Fecundity (calves • yr^{-1} • $female^{-1}$)	Female survival	Male Survival
1	0.000	0.880	0.880
2	0.000	0.940	0.940
3	0.009	0.970	0.970
4	0.060	0.970	0.970
5	0.250	0.970	0.970
6	0.440	0.970	0.970
7	0.491	0.970	0.970
8	0.499	0.970	0.970
9	0.500	0.970	0.970
10	0.500	0.970	0.970
11	0.500	0.972	0.969
12	0.500	0.972	0.969
13	0.500	0.972	0.969
14	0.500	0.972	0.969
15	0.500	0.972	0.969
16	0.500	0.972	0.969
17	0.500	0.972	0.969
18	0.500	0.972	0.969
19	0.500	0.972	0.969

20	0.500	0.972	0.969
21	0.500	0.972	0.969
22	0.500	0.972	0.969
23	0.500	0.972	0.969
24	0.500	0.972	0.969
25	0.500	0.972	0.969
26	0.495	0.972	0.969
27	0.490	0.972	0.969
28	0.485	0.972	0.969
29	0.480	0.972	0.969
30	0.475	0.972	0.969
31	0.470	0.967	0.963
32	0.465	0.961	0.957
33	0.460	0.954	0.949
34	0.455	0.946	0.940
35	0.450	0.936	0.930
36	0.445	0.926	0.918
37	0.440	0.913	0.904
38	0.435	0.897	0.887
39	0.430	0.880	0.867
40	0.425	0.859	0.844

41	0.420	0.834	0.817
42	0.415	0.806	0.785
43	0.410	0.772	0.748
44	0.405	0.732	0.704
45	0.400	0.686	0.653

Table S3: Detailed estimates of costs (2021 USD) associated with each management scenario

Method	Est. Cost (Col pesos)	Est. Cost (USD)	Expenses
Castration – in or near Nápoles	$40,000,000/hippo	$11,000/hippo	Capture and handling of hippo (expert vets, veterinary supplies, capture supplies)
Castration – outside Nápoles	$70,000,000/hippo	$19,000/hippo	Cost increases greatly due to skills needed by team members, as well as air transport in some places (helicopter US$ 8,200/hippo)
**Castration – all hippos**		**$12,813/hippo**	Assuming 58 hippos located near Nápoles and 17 located outside Nápoles
**Veterinary-assisted euthanasia – all hippos**	$40,000,000/1-2 hippos	**$7,333/hippo**	One month of work, which may cull 1-2 hippos; 2 professionals (10 million Colombian pesos); equipment and veterinary supplies (10 million Colombian pesos); transport and handling corpses (10 million Colombian pesos), transportation and other personal expenses (10 million Colombian pesos)

**Birth control in feed** (MGA) – only hippos in Nápoles	$10,275,000/hippo/year	**$2,800/hippo/year**	Mazuri ADF-16 0.5MA contains 0.5 mg MGA/lb. To get 6 mg of MGA, you would need 12 lbs. of feed per day per individual hippo you are feeding.<br><br>Can use cost estimates for Mazuri Wild Herbivore (5ZF1), which is $32 USD/50 lbs. (plus shipping costs)<br><br>Over a year, this would be 12lb/day*$0.64/lb*365 days/year = $2800/yr (plus shipping costs)<br><br>Because you are feeding them in a group of males and females, you would have to multiply this by the total number of captive hippos<br><br>This doesn't include extra costs to distribute feed to hippos outside of Nápoles area

Birth control in darts (Gonacon) – in or near Nápoles	$80,000,000/all hippos in Nápoles /year	$22,000/all hippos in Nápoles/year<br><br>$22,000/58 = $379/hippo/year	Includes one (1) professional, tools (rifles and darts) and medications; Approximate costs per year can be between 70 and 80 million Colombian pesos for a year of work; this would include professional salaries, transportation, rental or purchase of equipment, means of marking and purchasing veterinary supplies, for 12 months<br><br>NOTE: Updated estimates for only the cost of drugs is $10/hippo/dose, and there is a possibility that 3 doses will make a hippo sterile
Birth control in darts (Gonacon) – outside of Nápoles	$60,000,000/all hippos outside of Nápoles/year	$17,000/all hippos outside of Nápoles/year<br><br>$17,000/17 = $1000/hippo/year	1) Transportation - average costs for six months (3 million every month x six months = 18 million); 2) Professional for six months (monitoring and administration) (4 million/month x six months = 24 million); 3) Drugs and equipment (8-12 million; this may vary depending on the value of the medicines

			NOTE: Updated estimates for only the cost of drugs is $30/hippo/dart, and there is a possibility that 3 doses will make a hippo sterile
**Birth control in darts – all hippos**		**$520/hippo/year**	Assuming 58 hippos located near Nápoles and 17 located outside Nápoles

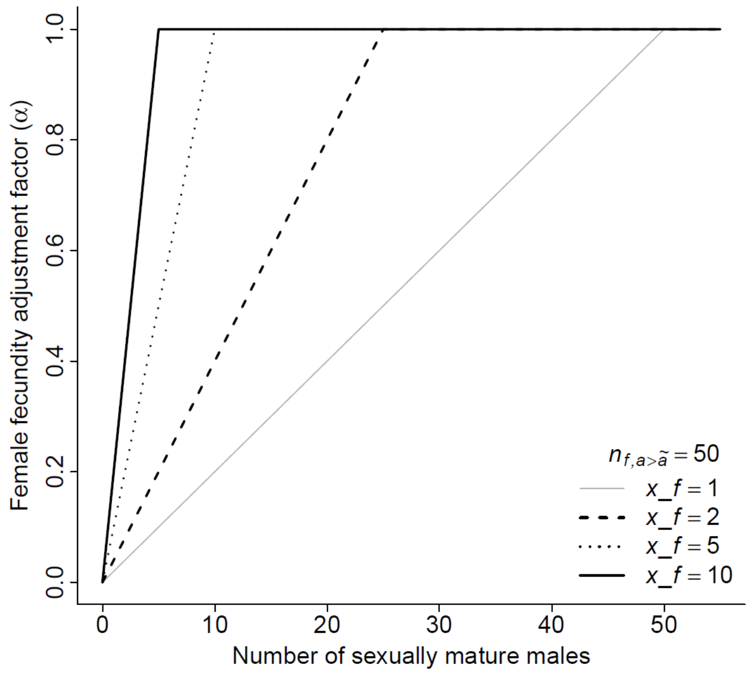

Figure S1. Female fecundity impacts associated with male hippo sterilization management interventions. Calculations show fecundity adjustment factors assuming a population of 100 sexually mature females and four different values for the harem size parameter, x_f, representing the number of females a single male can sire in a breeding season (eq. S4).
